# Non-invasive brain stimulation and cognitive function in patients with major depressive disorder or bipolar depression: systematic review and meta-analysis

**DOI:** 10.1192/j.eurpsy.2023.574

**Published:** 2023-07-19

**Authors:** J. Mutz, M. Kiebs

**Affiliations:** ^1^King’s College London, London, United Kingdom; ^2^University Hospital Bonn, Bonn, Germany

## Abstract

**Introduction:**

Non-invasive brain stimulation protocols are effective treatments for depressive episodes. Although the cognitive adverse effects of electroconvulsive therapy (ECT) are well documented, evidence regarding the cognitive effects of repetitive transcranial magnetic stimulation (rTMS) and transcranial direct current stimulation (tDCS) is mixed.

**Objectives:**

The aim of this study was to synthesize research on the cognitive effects of non-invasive brain stimulation protocols and to differentiate between studies of major depressive disorder (MDD), bipolar depression and mixed populations.

**Methods:**

Following a systematic literature search of multiple electronic databases, a series of meta-analyses were conducted to estimate standardized mean differences (SMD) between pre- and post-treatment cognitive functioning across nine cognitive domains. Where possible, SMDs were estimated separately for MDD, bipolar depression and mixed populations. In studies that included both patients with MDD and bipolar depression, the percentage of patients with a diagnosis of bipolar depression was tested as a potential moderator.

**Results:**

More than 150 treatment arms were included in the analyses. For ECT, we observed a small decline in language functioning and a decrease in autobiographical memory scores. There was no evidence of pre-post differences across other cognitive domains. For rTMS and tDCS, small to moderate cognitive improvements were observed for several cognitive domains, for example for executive functioning. Across most analyses, between-study heterogeneity was high and could not be accounted for by differences between MDD, bipolar depression or mixed populations.

**Conclusions:**

There was limited evidence that differentiation between studies of MDD, bipolar depression and mixed populations accounted for between-study heterogeneity in analyses of pre-post differences in cognitive functioning. Given that most studies included both patients with MDD and bipolar depression, this finding should be treated as preliminary. Across all the treatment protocols examined, more data are needed to investigate the cognitive effects of non-invasive brain stimulation in patients with bipolar depression.

**Disclosure of Interest:**

None Declared

